# Activity Regulation by Heteromerization of Arabidopsis Allene Oxide Cyclase Family Members

**DOI:** 10.3390/plants5010003

**Published:** 2016-01-06

**Authors:** Markus Otto, Christin Naumann, Wolfgang Brandt, Claus Wasternack, Bettina Hause

**Affiliations:** 1 Department of Cell and Metabolic Biology, Leibniz Institute of Plant Biochemistry, Weinberg 3, D-06120 Halle (Saale), Germany; mrmarkus.otto@googlemail.com (M.O.); christin.naumann@ipb-halle.de (C.N.); 2 Department of Molecular Signal Processing, Leibniz Institute of Plant Biochemistry, Weinberg 3, D-06120 Halle (Saale), Germany; cwastern@ipb-halle.de; 3 Department of Natural Product Chemistry, Leibniz Institute of Plant Biochemistry, Weinberg 3, D-06120 Halle (Saale), Germany; wbrandt@ipb-halle.de; 4 Laboratory of Growth Regulators, Centre of the Region Haná for Biotechnological and Agricultural Research, Institute of Experimental Botany AS CR & Palacký University, Šlechtitelů 11, CZ-78371 Olomouc, Czech Republic

**Keywords:** Arabidopsis allene oxide cyclase isoforms, heteromerization, protein structure analysis, site-directed mutagenesis, activity regulation

## Abstract

Jasmonates (JAs) are lipid-derived signals in plant stress responses and development. A crucial step in JA biosynthesis is catalyzed by allene oxide cyclase (AOC). Four genes encoding functional AOCs (AOC1, AOC2, AOC3 and AOC4) have been characterized for *Arabidopsis thaliana* in terms of organ- and tissue-specific expression, mutant phenotypes, promoter activities and initial *in vivo* protein interaction studies suggesting functional redundancy and diversification, including first hints at enzyme activity control by protein-protein interaction. Here, these analyses were extended by detailed analysis of recombinant proteins produced in *Escherichia coli*. Treatment of purified AOC2 with SDS at different temperatures, chemical cross-linking experiments and protein structure analysis by molecular modelling approaches were performed. Several salt bridges between monomers and a hydrophobic core within the AOC2 trimer were identified and functionally proven by site-directed mutagenesis. The data obtained showed that AOC2 acts as a trimer. Finally, AOC activity was determined in heteromers formed by pairwise combinations of the four AOC isoforms. The highest activities were found for heteromers containing AOC4 + AOC1 and AOC4 + AOC2, respectively. All data are in line with an enzyme activity control of all four AOCs by heteromerization, thereby supporting a putative fine-tuning in JA formation by various regulatory principles.

## 1. Introduction

The lipid-derived signaling compounds of the jasmonate family, among them jasmonic acid (JA) and octadecanoids, such as *cis*-(+)-12-oxophytodienoic acid (OPDA), are most active in plant stress responses and development. All enzymes involved in the biosynthesis of JA have been cloned and characterized from numerous plant species. Important components in JA perception and signaling have been elucidated (reviewed by [1]). Among them are repressors, such as the so-called JAZ (jasmonate ZIM domain) proteins and the F-box protein COI1 (coronatine insensitive1). Both of them are constituents of the SCF^COI1^ JAZ-co-receptor complex, which function as an E3 ubiquitin ligase (reviewed by [1]). This receptor has a regulatory role in JA biosynthesis, since genes encoding enzymes in JA biosynthesis are JA-inducible. Any proteasomal degradation of JAZ proteins will switch on expression of JA biosynthesis genes, thus being equivalent to a positive feedback loop in gene expression [2].

Since all enzymes of JA biosynthesis are constitutively present in most of the tissues, another level of regulation takes place by substrate availability [1]. α-Linolenic acid (α-LeA) is the initial substrate in the biosynthesis of JA, which is only generated upon the release of α-LeA from chloroplast membranes. Such release takes place by environmental stimuli, as wounding, or during development. The requirement for substrate generation was shown by transgenic lines overexpressing enzymes in JA biosynthesis [3,4]. Such lines contain high levels of biosynthetic proteins, but do not show enhanced basal levels of JA. However, upon stimulus leading to release of α-LeA, JA is synthesized to higher levels than in wild-type plants. The tissue specificity of the occurrence of JA biosynthesis enzymes is an additional regulatory level. The tissue-specific and common occurrence of enzymes is attributed to the capacity of different cells and tissues for JA formation and JA signaling [4,5,6,7]. Finally, post-translational regulation is suggested to be attributed to the regulation of JA biosynthesis since the first hints at protein-protein interaction among the biosynthetic enzymes were obtained [8,9]. Moreover, employing labelled JA or the synthetic JA-Ile mimic coronalon to distinguish exogenous and endogenous jasmonates, no jasmonate biosynthesis/accumulation could be found supporting a post-translational regulation [10,11].

JA biosynthesis takes place by a sequential action of a 13-lipoxygenase (13-LOX), 13-hydroperoxide-converting 13-allene oxide synthase (13-AOS), cyclopentenone-forming allene oxide cyclase (AOC), the cyclopentanone-forming OPDA reductase3 (OPR3), as well as the fatty acid β-oxidation machinery, including an acyl-CoA-oxidase (ACX1), in the shortening of the carboxylic acid side chain. In Arabidopsis, these enzymes are encoded by single copy genes (AOS, OPR3) or by small gene families (13-LOX, AOC, ACX1). In case the enzymes are active as multimers, such gene families open the possibility for regulation by multimerization, if all or several isoforms are present in the same tissue or compartment. The first indication for such a regulation has been described for OPR3 and AOC, which were shown to be, upon crystallization, a dimer [8] and a trimer [12], respectively. These studies analyzed predominantly the binding pocket of OPR3 and AOC2, respectively. Less is known, however, for the structural requirement of trimerization of AOC2 and putative heteromerization of AOCs. The *AOC* gene family in Arabidopsis comprises four members, which encode functional AOC1, AOC2, AOC3 and AOC4 in a COI1- and JA-dependent manner, including wound-induced expression [13]. For the four *AOC* promoters, redundant, as well as organ- and isoform-specific activities have been detected [9]: (i) in roots, only *AOC3* and *AOC4* showed promoter activity; (ii) in fully-developed leaves, *AOC1*, *AOC2* and *AOC3* promoters were active, whereas the *AOC4* promoter activity was confined to the vascular bundles; (iii) *AOC1* and *AOC4* promoter activities were found to be partially specific in flower development. Single and double *AOC* loss of function mutants were without any altered phenotype, further supporting the assumption of redundancies among the AOCs of Arabidopsis. Analyses by bimolecular fluorescence complementation (BiFC) indicated *in planta* interaction of all four AOCs to each other [9]. Whether this interaction of different isoforms results in altered enzymatic activities could, however, not be clarified.

Here, we complement and extend this work by analysis of the trimer formation of AOC2 and addressed the question of whether heteromers between different AOC isoforms do show altered enzymatic activities. The formation of AOC2 trimers has been analyzed by using selective treatments with sodium dodecyl sulfate (SDS) and chemical cross-linking, as well as by molecular modelling studies, leading to the identification of salt bridges and a hydrophobic core, which have been proven by site-directed mutageneses. Finally, the activities of recombinantly-expressed AOC heteromers were determined in comparison to the activity of homomers. The data are in line with an enzyme activity control of AOCs by heteromerization, thereby supporting a fine-tuning in the formation of JAs by an additional regulatory principle.

## 2. Results

### 2.1. Trimer Formation of Recombinant AOC2

**Figure 1:**
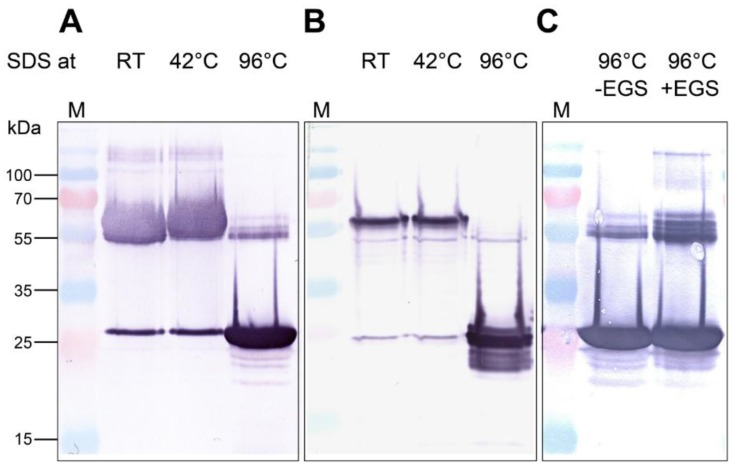
Recombinant Arabidopsis allene oxide cyclase 2 (AOC2) is a trimer. His-tagged proteins were purified on Ni-NTA, treated with SDS at given temperatures for 5 min and separated by SDS-PAGE. Detection of proteins was performed by immunolabeling using an anti-His-tag antibody. (**A**) Recombinant AOC2 protein with N-terminal His-tag; (**B**) recombinant AOC2 protein with C-terminal His-tag; (**C**) recombinant AOC2 protein with N-terminal His-tag cross-linked or not with 200 μM glycol-bis(succinic acid *N*-hydroxysuccinimide ester) (EGS) for 40 min. Note that only treatment at 96 °C resulted in the predominant occurrence of monomers (~26 kDa), whereas trimers resisted treatments at lower temperatures. Accordingly, cross-linking with EGS prevented separation of trimers by SDS treatment at 96 °C (C). M = size marker.

The AOC2 has been determined as a trimer in crystals, and SDS-stable trimer formation was suggested for recombinantly-expressed AOC exhibiting an N-terminal His-tag, whereas the C-terminally tagged AOC2 appeared in monomers only [12]. To clarify the role of AOC2 trimer formation, we extended here these analyses. *AOC2* was fused N- and C-terminally to His-tag and expressed in *E. coli*. After purification of AOC2 using Ni-nitrilotriacetic acid (Ni-NTA), proteins were treated with SDS at room temperature, 42 °C and 96 °C for 5 min and separated by SDS-polyacrylamide gel electrophoresis (PAGE) (Figure 1A,B). After treatment at room temperature and 42 °C, most of the recombinant AOC2 was found at around 65 kDa, pointing to the appearance as a trimer independently on the site of the His-tag. After treatment at 96 °C, however, AOC2 was mostly in the monomeric form, exhibiting the expected size of 26 kDa. Lastly, solutions containing AOC2 were incubated with the chemical cross-linking agent ethylene glycol-bis(succinic acid *N*-hydroxysuccinimide ester) (EGS) and analyzed after treatment with SDS at 96 °C. In these experiments, EGS, as a zero-length spacer, forms covalent intermolecular bonds [14] between subunits in AOC2 multimers, allowing definitive identification of the subunit structure(s) of AOC2 in the presence or absence of detergent (Figure 1C). Cross-linking with EGS led to an increase in the amount of AOC2 in the trimeric state, which was not dissolved by treatment with SDS at 96 °C. This was also supported by size exclusion chromatography (Figure S1). These data confirm that the predominant form of recombinant, His-tagged AOC2 is a trimer.

### 2.2. Analysis of the Quaternary Structure of AOC2

**Figure 2:**
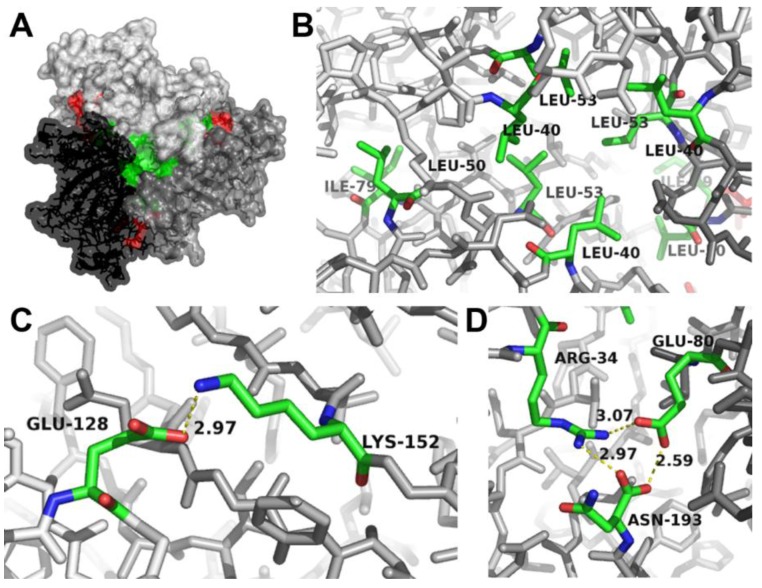
Identification of amino acids at the interaction sites between AOC2 monomers within the trimer. (**A**) Surface representation of the AOC2 trimer (PDB Code 2Q4I). The view is along the trimer axis. The three monomers are given in different gray scales. The hydrophobic core (detailed in (**B**)) and the salt bridges (detailed in (**C**)) are shown in green and red, respectively. Salt bridges on the back are detailed in (**D**); see Figure S1. (B) Location of Leu40, Leu50, Leu53 and Ile79 of all three monomers building the hydrophobic core of the trimer. (C) Salt bridge (yellow dashed line) between Lys152 of one monomer and Glu128 of the neighboring monomer. The distance between atoms building the salt bridge is given in Å. (D) Hydrogen bonds between Arg34 and Asn193 of one monomer and salt bridges (yellow dashed lines) to Glu80 of the neighboring monomer. The distances between atoms building a salt bridge are given in Å. Interacting faces are shown in stick representation (oxygen, red; nitrogen, blue; carbon, green; atoms not involved in the interaction, gray).

To clarify whether the formation of AOC2 trimer is essential for enzymatic activity, the structure of the trimer was analyzed by molecular modelling methods, including determination of activity after site-directed mutagenesis. For the modelling approach, the crystal structure of AOC2 available from the Protein Data Bank and deposited as 2Q4I was used (Figure 2A). This structure resulted from an improved refinement protocol using the original data of the Center for Eukaryotic Structural Genomics (CESG) and showed the most consistent R-free values at high resolution. The calculation of the interaction energies of all single monomers (chains A–C) from the remaining dimers (*i.e.*, the interactions of chain A with the dimer consisting of chains B and C and all other combinations) yielded in average −250 kcal/mol (±5 kcal/mol). This rather high interaction energy is a strong indication of the stability of the trimer complex, such as crystallized.

Inspection of the contact surfaces between the different chains showed the formation of hydrophobic interaction sites building a hydrophobic core (Figure 2A,B). The hydrophobic core is formed by the amino acids Leu40, Leu50, Leu53 and Ile79 of each monomer. In addition, different salt bridges between the monomers were found and are located on both sites of the trimer (Figure 2A and Figure S2). On the one hand, strong hydrogen bonds were detected between the side chains of Arg34 and Asn193 of one monomer and a salt bridge with Glu80 of the neighboring monomer (Figure 2C). These interactions have been identified previously by Hofmann *et al.* [12], but due to the other crystal structure used (PDB Code 2BRJ), the involved amino acids are numbered there as Arg29, Asn188 and Glu75. On the other hand, additional salt bridges were identified on the “back-side” of the trimer (Figure S1) and are formed between Glu128 of one monomer and Lys152 of the neighboring monomer (Figure 2D).

**Figure 3:**
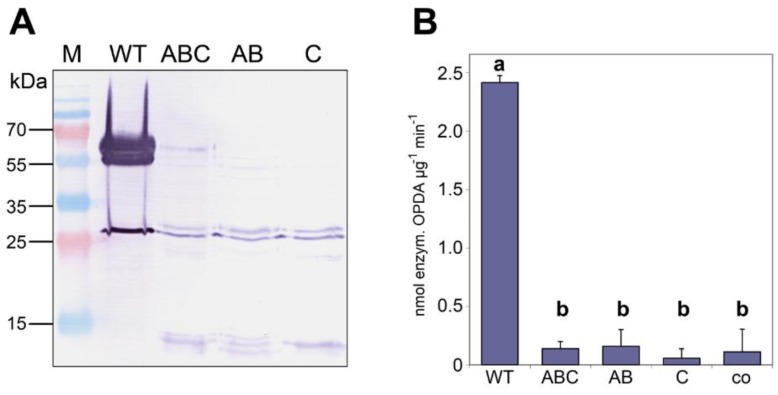
Monomers of AOC2 do not exhibit enzymatic activity. AOC2 was mutated to prevent trimer formation by the exchange of amino acids involved in salt bridge formation (K152A/E80A = “AB”), the formation of the hydrophobic core (L53S = “C”) or both (K152A/E80A/L53S = “ABC”) (see Table 1). (**A**) His-tagged recombinant proteins were treated with SDS at 42 °C for 5 min and separated on SDS-PAGE. Note that all mutant proteins are predominantly detectable as monomers. M = size marker. (**B**) AOC enzyme activity of recombinant wild-type AOC2 and mutant proteins. Each value is given as nmol of enzymatically-formed *cis*-(+)-12-oxophytodienoic acid (OPDA) per μg protein and min and is represented by the mean of three independent replicates ± SD. Different letters designate statistically-different values (one-way ANOVA with Tukey’s HSD test, *p* < 0.01). co = control done without the addition of protein to the reaction mixture.

To test whether monomeric AOC2 exhibits enzymatic activity, the identified interaction sites were mutated to prevent the formation of a trimeric quaternary structure (Table 1). All three mutants and the AOC2 wild-type form were expressed as His-tagged versions in *E. coli* and purified (Figure 3A), whereby the yield of purified proteins was very low in the case of all mutant versions. The question could not be clarified, however, why there was a diminished production of recombinant proteins. Nevertheless, the purified mutant proteins did not appear different from wild-type AOC2 in regard to stability. Therefore, the quaternary state was compared upon SDS treatment of AOC2 proteins at 42 °C for 5 min (Figure 3A). Whereas the wild-type protein is clearly visible as a trimer with an apparent mass of about 65 kDa, the mutated AOC2 variants are monomeric. All purified proteins were used in equal amounts (10 μg purified protein each) to determine enzymatic activity by using the coupled enzymatic test by incubation with recombinant HvAOS [15] and 13(*S*)-hydroperoxyoctadeca-9,11,15-trienoic acid (HPOT) as the substrate. The production of *cis*-OPDA was recorded as enzymatically-formed OPDA and was measured in high amounts for wild-type AOC2 (Figure 3B). In contrast, proteins mutated in amino acid residues involved in the formation of salt bridges, in the hydrophobic core, or in both, reduced the AOC2 activity dramatically. Here, the detectable amount of *cis*-OPDA did not exceed that of the control performed by omitting any AOC protein. All of these data are in line with an essential role of trimer formation for AOC2 activity.

**Table 1:** Mutants of Arabidopsis AOC2 and expected alterations in the interactions of monomers.

Mutant	Exchange of Amino Acids	Expected Effects
AB	K152A, E80A	Disruption of salt bridges
C	L53S	Disruption of hydrophobic core
ABC	K152A, E80A; L53S	Disruption of salt bridges and hydrophobic core

### 2.3. Heteromerization of AOC Gene Family Members and Its Effect on AOC Activity

The trimer formation of AOC2 raises the question of whether the activity of AOCs might be affected by putative heteromerization of different AOCs. The formation of heteromers between AOC family members has been detected upon *in planta* protein interaction studies using BiFC [9]. Even if interactions among all AOCs were found, the effect on activity by an *in planta* proof was, however, masked by overexpression conditions [9].

**Figure 4:**
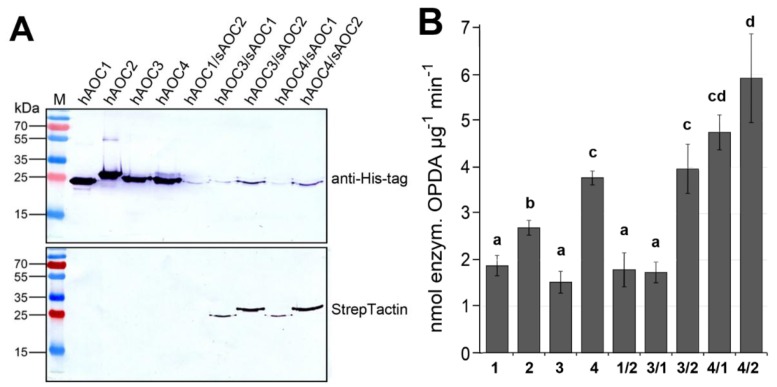
Heteromerization of AOC results in altered activities. (**A**) Purification of His-tagged homomers was done using Ni-NTA (left). Heteromers were purified using Ni-NTA (for His-tag) and StrepTactin (for Strep-tag) subsequently. Immunoblots of purified recombinant proteins treated with SDS at 96 °C show homomers with His-tag (hAOC) and heteromers exhibiting one isoform with His-Tag (hAOC) and the other with Strep-tag (sAOC). Note that the heteromers were detectable by both immuno-decorations. (**B**) Activity of recombinant homomeric and heteromeric AOCs. Each value is given as nmol of enzymatically-formed OPDA per μg protein and min and is represented by the mean of three independent replicates (±SD) obtained from independent protein preparations. Different letters designate statistically-different values (one-way ANOVA with Tukey’s HSD test, *p* < 0.05).

To check the influence of heteromerization on AOC activity, we purified the four recombinant AOCs as homomers using N-terminally His-tagged versions expressed in *E. coli*. To get heteromers, different pairwise combinations of all AOCs were expressed using one vector, thereby one AOC marked by a His-tag and the other AOC with a Strep-tag (Figure 4A). Heteromers were purified first using Ni-NTA for the His-tag followed by StrepTactin for the Strep-tag. After treatment with SDS at 96 °C and separation by SDS-PAGE, the homomers and heteromers of the AOCs were detected by immunoblot analyses (Figure 4A). The His-tag-labeled homomers of the four AOCs (Figure 4A, top left) were clearly purified, exhibiting slightly different molecular masses according to previously-published results [13]. Purification of the heteromers carrying His-tagged and the Strep-tagged monomers is indicated by their appearance in immunoblot analysis with both tag-specific antibodies (Figure 4A, right, top and bottom). All of these purified protein variants were used for the determination of activity (Figure 4B). The AOC activities of homomeric and heteromeric AOCs showed significant differences. Among the homomers, AOC4 exhibited the highest activity, whereas AOC1 and AOC3 showed similar, but low activity. Most importantly, the activities of several heteromeric AOCs exceeded that of homomeric AOCs. The highest activity was found for heteromers of AOC4 and AOC2, as well as AOC4 and AOC1 (Figure 4B). These data strongly suggest that AOC activity is altered by heteromerization of the AOC family members.

## 3. Discussion

In the biosynthesis of jasmonates, AOC is of crucial importance, since it establishes the enantiomeric structure of the cyclopentenone ring. Crystal structures for AOCs from Arabidopsis [12,16,17] and *Physcomitrella patens* [18] have been obtained and revealed mechanistic information on catalysis, but additionally showed that these AOCs form a trimeric quaternary structure. Among the four isoforms occurring in Arabidopsis, AOC2 has been described as the most active form and has been intensively studied in terms of structure-function analysis regarding the active center [19]. The main structural feature of the AOC2 monomer is the central eight-stranded antiparallel β-barrel. The active site is located inside the barrel cavity and is mostly lined by hydrophobic and aromatic amino acids that help to coordinate the positioning of the substrate. The stereospecific catalysis was found to be a direct result of isomerization of the substrate *cis*-12,13*S*-epoxy-9*Z*,11*Z*,15*Z*-octadecatrienoic acid (12,13-EOT) due to this protein environment [19]. Within the crystallized trimer, the barrel axes of the monomers are tilted ~30° with respect to the trimer axis and pack closely with their barrel walls [12]. However, the question of whether the formation of trimeric structures is essential for AOC activity and how heteromers formed by different isoforms could affect the activity was not addressed yet.

### 3.1. Recombinant AOC2 of Arabidopsis Is Active as a Trimer

To better understand the AOC2 multimerization, we inspected the trimer formation of recombinant AOC2 of *A. thaliana* with respect to its activity. The oligomeric state of AOC2 was examined in the presence of detergent at various temperatures followed by gel-electrophoresis. We could not find any influence of the location of His-tag on the trimeric state of the protein, and the treatments with detergent and analyses by cross-linking using EGS supported the occurrence of the trimer as the predominant form of AOC2. This is in line with the crystallization data, but also with the detection of AOC2 from plant extracts in SDS PAGE or immunoblots, although these statements were given without data presentation [12].

Calculation of the interaction energy between the monomers strongly supported the stability of the trimer. Two sites at each monomer that form salt bridges and a hydrophobic core built by Leu40, Leu50, Leu53 and Ile79 of each monomer were found (Figure 2). All amino acids involved in this interaction are located apart from the hydrophobic binding pocket for the substrate. The trimer formation seems to be essential for activity, since site-directed mutagenesis of amino acid residues involved in trimer formation led to nearly complete loss of activity (Figure 3). This is in contrast to Hofmann *et al.* [12], where trimer formation was not assumed to be required for activity, but suggested for the improvement of overall protein stability. Interestingly, residues involved in the interaction between the monomers are predominantly conserved in all known AOC sequences [19], and AOC1 from Arabidopsis (PDB Code 1ZVC) and AOC1 and AOC2 from *P. patens* [18] crystallize as a trimer, as well. This supports the hypothesis that the trimer represents a relevant quaternary structure of enzymatically-active AOCs.

### 3.2. Heteromers between Different AOC Isoforms Exhibit Altered Activity

The four members of the AOC gene family of *A. thaliana* exhibit a redundant expression pattern, as also shown by promoter activity analyses [9]. Such a redundant expression pattern raises the question of the putative heteromerization of the various AOC isoforms. Indeed, the BiFC analysis showed an *in vivo* interaction of all AOCs with each other [9]. The *in planta* proof for the effects on activity done by monitoring the wound-induced transcript accumulation of a JA-responsive gene, however, did not deliver unequivocal results. *In planta*, homomers and heteromers could be formed simultaneously, leading to an overall high level of AOC protein and, thereby, overriding the putative effect of activity control. Consequently, putative heteromerization was analyzed here by pairwise combination of the four recombinantly-produced isoforms. This system allows the determination of the activity of the single AOC monomers and combinations of heteromers without effects by endogenous plant proteins.

Heteromer formation could be clearly documented in immunoblot analysis via detection of His-tag and Strep-tag, respectively (Figure 4A). The significant differences among the AOC activities of heteromeric combination strongly suggest the influence of heteromerization on AOC activity (Figure 4B). The highest activity was detected for heteromers of recombinant AOC4 together with AOC1 or with AOC2. This finding is further underscored by *in vivo* interaction studies of Arabidopsis AOCs, where preferentially, these pairs showed the strongest signals in the BiFC analyses [9]. Moreover, promoters of *AtAOC4* and *AtAOC2* are both active in the vasculature of mature leaves [9], the tissue that is most important for the production of jasmonates upon wounding [20]. Furthermore, promoters of *AtAOC4* and *AtAOC1* are commonly active in filaments of stamen [9]. In filaments, biosynthesis of JA is of special importance, since JA-deficient and -insensitive mutants are male sterile, showing a defect in filament elongation [21].

The mechanism on which the activity shift is based, however, remains unclear. The amino acid sequences of all four AOCs are highly similar [13], with all amino acids postulated to be involved in the cyclization reaction being conserved [12]. Therefore, at least the three-dimensional structure of their monomers, including the substrate-binding pocket, appears to be nearly identical [17]. Substrate binding within the active site does not involve an induced fit mechanism [17], since it is facilitated by the hydrophobic protein environment, as described above. Therefore, it is questionable whether the rigid barrel might be changed in its structure upon the interaction between different monomers. Nevertheless, despite their similarity, already, all four AOCs of Arabidopsis convert their substrate with different efficiencies, with the highest activity for AOC4 and the lowest activities for AOC1 and AOC3 (Figure 4).

## 4. Experimental Section

### 4.1. Cloning, Mutagenesis, Recombinant Expression and Purification of AOCs

cDNAs of *AtAOC1* (At3g25760), *AtAOC2* (At3g25770), *AtAOC3* (At3g25780) and *AtAOC4* (At1g13280) were used without the plastid signal sequence [13]. Mutated versions of AOC2 were created *in silico*, and DNA was synthesized by GENEART (Thermo Fisher Scientific, Dreieich, Germany, www.thermofisher.com). All constructs were introduced into pQE30 (N-terminal His-tag) or pQE60 (C-terminal His-tag). To obtain heteromeric versions, different combinations of two wild-type AOCs were cloned together into pQE30, whereby one AOC was tagged with His-tag, and the other AOC was N-terminally tagged with Strep-tag-II. All plasmids were transformed into the host strain *E. coli* M15. The total protein of isopropyl-β-thiogalactopyranoside (IPTG)-induced cultures was isolated, and AOC proteins were purified using Ni-NTA-agarose (GE Healthcare, Munich, Germany, www.gehealthcare.com) for His-tagged versions, as described [22]. To purify the heteromeric combinations of AOCs, the eluate from Ni-NTA-agarose was directly used for the second purification step. Here, StrepTrap HP (GE Healthcare) was applied to purify Strep-tagged proteins according to the manufacturer’s instructions. The amount of all purified proteins was determined by the bicinchoninic acid (BCA) assay (Sigma-Aldrich, Taufkirchen, Germany, www.sigmaaldrich.com). Equal amounts of proteins were used directly for SDS-PAGE and the determination of activity, as described below.

### 4.2. Structure Analysis and Modelling of AOC Trimers

The X-ray structure of the AOC2 from *Arabidopsis thaliana* (At3g25770, PDB Code 2Q4I, [16]) was used for the modelling calculations. This structure was crystallized as a homo-trimer. To gain insight into the stability of the homo-trimeric structure, interaction energies between a monomer with the remaining dimer have been calculated. For this purpose hydrogen atoms were added to the X-ray structure with the help of MOE (Molecular Operating Environment Version 2014.09, chemical computing Group, Cologne, Germany, https://www.chemcomp.com) using the protonate 3D module. Subsequently, the homo-trimer structures were energy optimized using the CHARMM27 force field [23] with Born solvation [24]. The interaction energies of each monomer with the remaining dimer were calculated by subtracting the force field energies of the mono and dimer structures from the energy of the entire trimer. Additionally, the structure of AOC2 was analyzed using Pymol (www.pymol.org). To determine non-covalent interactions between the subunits of the homo-trimeric structure, electrostatic interactions were addressed between oppositely-charged amino acids Asp or Glu with Arg, Lys or His (salt bridges) and increased abundance of hydrophobic amino acids (Leu, Ile, Val) on the surface of the interacting monomers. To validate the interaction between promising candidates, measurements were performed using the software Pymol. A salt bridge was defined as an ion pair, if the centroids of the side-chain charged-group atoms in the residues lie within 4.0 Å of each other and at least one pair of Asp or Glu side-chain carbonyl oxygen and side-chain nitrogen atoms of Arg, Lys or His are also within this distance [25].

### 4.3. Cross-Linking, Size Exclusion Chromatography, SDS-PAGE and Immunoblot Analysis

The cross-linking reagent, ethylene glycol-bis(succinic acid *N*-hydroxysuccinimide ester) (EGS), was purchased from Sigma–Aldrich. Purified AOC2 protein was treated with 200 μM EGS for 40 min at room temperature. The reaction was stopped by adding 0.1 volumes of 1 M Tris-Cl/1 M glycine (pH 7.5). Cross-linked proteins, as well as AOC2 proteins directly after purification were separated on a HiLoad 16/60 Superdex 200 prep grade column (17-1069-01, GE Healthcare Life Science, Freiburg, Germany, www.gelifesciences.com) using an Äkta Explorer System (GE Healthcare Life Science). The molecular weight of fractions was calculated using a calibration curve (see Figure S1), whereby dextran blue (MW 2000 kDa) was used to obtain v_o_. For SDS-PAGE, proteins were treated with 1% sodium dodecyl sulfate (SDS) at different temperatures for 5 min and separated on a 12% polyacrylamide gel [26]. Immunoblot analyses using an anti-His-tag antibody from mouse (C 0409, Novagen, Darmstadt, Germany, www.merckmillipore.com) or StrepTactin coupled to horseradish peroxidase (IBA Bio*tag*nology, Goettingen, Germany, www.iba-lifesciences.com) were performed as described [13].

### 4.4. Determination of AOC Activity

AOC activity was determined according to [27] with modifications described in [28]. Briefly, 10 μg of purified recombinant proteins were incubated with recombinant HvAOS [15] and 13(*S*)-hydroperoxyoctadeca-9,11,15-trienoic acid (HPOT) at 4 °C for 10 min. The reaction was stopped by acidification, and Me-OPDA was added as the internal standard. Extraction with diethyl ether and evaporation of extract was performed followed by treatment with 0.2 M NaOH (in methanol) to activate trans-isomerization of *cis*-(+)-OPDA. After incubation at 4 °C for 60 min, the reaction was stopped by neutralization with 2 N HCl. The reaction mixtures were extracted with 2 mL of diethyl ether, evaporated and subjected to chiral phase HPLC, as described. The absolute content of OPDA was calculated using the internal standard. The percentage of enzymatically-formed *cis*-OPDA was calculated according to [29].

## 5. Conclusions

The regulation of enzyme activity by heteromerization is a repeatedly observed property of enzymes. A prominent example in plant hormone biosynthesis is ACC synthase, where different combinations of nine isoforms are attribute to altered ethylene formation [30,31]. The altered AOC activity by the heteromerization of members of the AOC family can be regarded as an additional regulatory principle in jasmonate biosynthesis, where substrate generation, posttranslational modifications and tissue specificity were discussed so far to have a regulatory role [1,11,32,33].

## Supplemental Materials

Figure S1: Determination of the multimerization state of AOC2 by size exclusion chromatography (SEC). Figure S2: Surface representation of the AOC2 trimer (back-side of Figure 1A). They can be accessed at: http://www.mdpi.com/2223-7747/5/1/3/s1.
