# Supplementary material for: Activity Regulation by Heteromerization of Arabidopsis Allene Oxide Cyclase Family Members
Source: Plants (Basel). 2016 Jan 6;5(1):3. doi: 10.3390/plants5010003 (PMC4844422; doi:10.3390/plants5010003)
Supplement: Supplementary file 1 [file plants-05-00003-s001.pdf]

Supplementary Materials

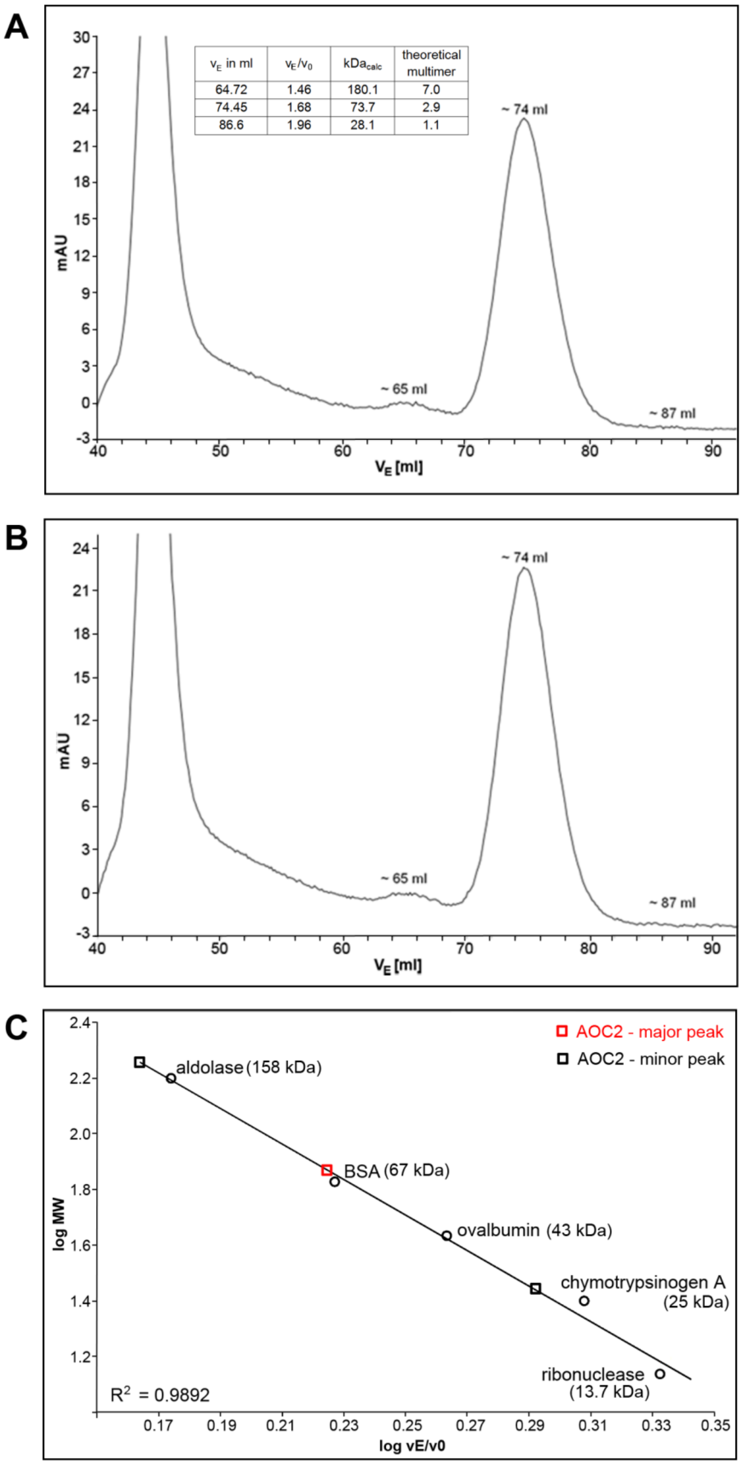

Figure S1. Determination of multimerization state of AOC2 by size exclusion chromatography (SEC). Recombinantly produced AOC2 was purified using His-tag. One mg of protein was either directly separated (**A**) or cross-linked using EGS before separation (**B**) on HiLoad 16/60 Superdex 200 prepgrade column. Note that two peaks were obtained, from which the peak at the elution volume of about 74 ml represents the majority of AOC2. The calculation of the molecular weight including of the number of monomers is given in the insert in A. The determination of apparent molecular weight of the three AOC2 fractions was done using calibration line (**C**) obtained by separation of proteins with known molecular weights (aldolase, BSA, ovalbumin, chymotrypsimogen A and ribonuclease). Position of AOC2 hexamers, trimers and monomers, respectively, is indicated by squares.

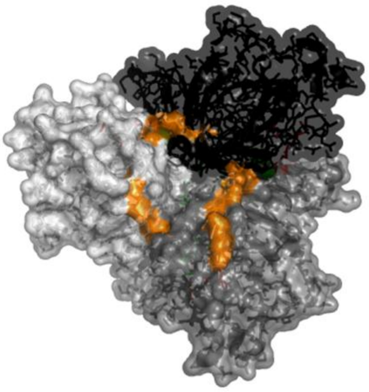

Figure S2. Surface representation of the AOC2 trimer (back side to Figure 1A). The view is along the trimer axis. The three monomers are given in different gray scales. The salt-bridges are shown in orange.
